# Intestinal barrier dysfunction in severe burn injury

**DOI:** 10.1186/s41038-019-0162-3

**Published:** 2019-07-26

**Authors:** Wen He, Yu Wang, Pei Wang, Fengjun Wang

**Affiliations:** 1 State Key Laboratory of Trauma, Burns, and Combined Injury, Institute of Burn Research, Southwest Hospital, Third Military Medical University (Army Medical University), Chongqing, 400038 China; 2 Department of Gastroenterology, Southwest Hospital, Third Military Medical University (Army Medical University), Chongqing, 400038 China

**Keywords:** Burn, Intestinal barrier dysfunction, Tight junction, Myosin light chain, Myosin light chain kinase, Rho-associated protein kinase

## Abstract

Severe burn injury is often accompanied by intestinal barrier dysfunction, which is closely associated with post-burn shock, bacterial translocation, systemic inflammatory response syndrome, hypercatabolism, sepsis, multiple organ dysfunction syndrome, and other complications. The intestinal epithelium forms a physical barrier that separates the intestinal lumen from the internal milieu, in which the tight junction plays a principal role. It has been well documented that after severe burn injury, many factors such as stress, ischemia/hypoxia, proinflammatory cytokines, and endotoxins can induce intestinal barrier dysfunction via multiple signaling pathways. Recent advances have provided new insights into the mechanisms and the therapeutic strategies of intestinal epithelial barrier dysfunction associated with severe burn injury. In this review, we will describe the current knowledge of the mechanisms involved in intestinal barrier dysfunction in response to severe burn injury and the emerging therapies for treating intestinal barrier dysfunction following severe burn injury.

## Background

It is well known that the polarized epithelial cells of intestinal mucosa form a barrier to prevent luminal pathogens and antigenic molecules from entering the intestinal mucosa and contacting with the immune system, to which the tight junction (TJ) and its associated proteins are critical. However, the intestinal epithelial barrier disruption often develops in many diseases including severe burn injury. The severe burn-induced disruption of intestinal barrier results in the increased intestinal permeability and subsequent translocation of bacteria and/or endotoxin from the gastrointestinal tract to cause systemic inflammatory response syndrome, sepsis, multiple organ dysfunction syndrome, and other critical complications. Previous studies have shown a close relationship between intestinal barrier disruption and the incidence and severity of sepsis in critically ill patients [1, 2]. It has been demonstrated that 60% of critically ill patients suffering from multiple organ failure develop intestinal barrier disruption [2]. The burn victims occurring intestinal barrier disruption are at high risk for bacterial translocation, sepsis, and mortality [3]. Thus, the intestinal barrier disruption may play a central role in the development of multiple critical complications elicited by severe burn injury. Although the underlying mechanisms of intestinal barrier disruption induced by severe burn injury are not well understood, intensive research efforts regarding post-burn intestinal barrier dysfunction have been ongoing. The overall goal of this review is to describe the intestinal barrier disruption in severe burn injury, with a focus on the potential molecular mechanisms.

## Review

### Overview of intestinal barrier and TJ

The basic and important function of the intestinal tract is to digest and absorb nutrients. In addition, the intestinal tract is not only the largest immune organ in the body, but also the largest reservoir of bacteria and endotoxin. Under physiological condition, the intestinal mucosa allows only small molecules to pass, which relies on the intact mucosa barrier to effectively prevent luminal bacteria, endotoxins, and other antigens from translocating to other distant organs [4–6]. The intestinal barrier includes mechanical, immunological, biological, and chemical barriers. The mechanical barrier is mainly composed of the mucous layer on the surface of the intestinal mucosa, intestinal epithelial cells, intercellular junctions, submucosal lamina propria, etc. If there is no specific transporter, most hydrophilic solutes cannot permeate the mechanical barrier [5, 6]. The immunological barrier, which mainly consists of a large number of immunocompetent cells, including lymphocytes, macrophages, dendritic cells, and plasma cells, has the functions of antigen presentation, inflammatory mediators secretion, and mucosal allergic response [7]. The secretory IgA-mediated humoral immune response plays an important role in the intestinal immunological barrier. *Lactobacillus* and *Bifidobacterium*, which are the intestinal resident bacteria, mainly form the biological barrier. The resident bacteria form biofilm on the intestinal epithelial surfaces, resist the invasion of exogenous pathogenic bacteria, and provide the intestinal epithelial cells with nutrients by producing short-chain fatty acids, lactic acids, and others. The chemical barrier is the generic name for the antibacterial substances produced by the resident intestinal bacteria and chemical substances such as gastric acid, bile, digestive enzymes, muramidase, and mucopolysaccharide, which can inhibit the adherence and colonization of bacteria [8].

The mechanical barrier is a pivotal part of the intestinal barrier and maintained through the intestinal epithelial cells and intercellular junctions [4–6]. The intercellular junctions comprise the tight, gap, adhesion, and desmosome junctions. The TJ, consisting of multiple proteins such as zonula occludens (ZO), occludin, the claudins, and the junctional adhesion molecules (JAM), is a complex that forms a selectively permeable seal between adjacent epithelial cells and serves as a selective barrier between the plasma membranes of adjacent cells [9–11]. The TJ is in a state of relatively stable dynamic remodeling and regulated by various factors such as Ca^2+^-E-cadherin, Rho-GTPase, phospholipase C, protein kinase A, tyrosine kinase, mitogen-activated protein kinases (MAPK), and myosin light chain kinase (MLCK). Among these factors, MLCK plays a critical role in the regulation of TJ dynamic. By regulating the phosphorylation of myosin light chain (MLC), MLCK is associated with the perijunctional actomyosin ring [5].

Occludin, belonging to transmembrane proteins, is composed of four transmembrane domains, two extracellular domains, and a long cytoplasmic C-terminal tail [12]. The intracellular C-terminus of occludin is connected with ZO. Occludin has important significance in maintaining the paracellular permeability and transepithelial electricity [13–15]. The function of occludin depends on the phosphorylation of serine and threonine residues. The small GTPase- and protein kinase c (PKC)-related signaling pathways are involved in the regulation of occludin phosphorylation.

The claudin family contains more than 20 members, which have an extracellular loop structure of variable amino acid residues and an intracellular short tail structure [16–18]. Different subtypes of claudins are expressed in different tissues and cells. Claudins are not only the barrier-forming components of the TJ. There are also pore-forming claudins including claudin-2 and claudin-10. Thus, claudins play a critical role in the physical regulation of paracellular permeability by forming the intercellular ion channel [16].

ZO is a member of the so-called membrane-associated guanylate kinase (MAGUK) family and has the isomers ZO-1, ZO-2, and ZO-3 [19–23]. ZOs contain conserved sequences such as guanylate kinase (GUK) structure domains, PDZ domains, the Src-homologous SH3 domain, the acidic domain, the arginine-rich domain and the proline-rich domain, and these conserved sequences take part in the connection between ZO and other proteins. For example, ZO directly binds to the C-terminus of claudins through possynaptic density protein-95/Discs-Large/zonula occludens-1 (PDZ) domain, which initiates and/or facilitates the polymerization of claudins and is crucial for the formation of TJs [20]. These diverse interactions determine that ZO-1 has a scaffolding function. The JAMs are glycoproteins belonging to the immunoglobulin supergene family, which consists of two extracellular immunoglobulin-like structures, a transmembrane domain, and an intracellular region with PDZ binding sequence. The JAMs have multiple functions including the regulation of intestinal epithelial paracellular permeability [24, 25].

### Characteristics of intestinal barrier dysfunction in severe burn injury

**Fig. 1:**
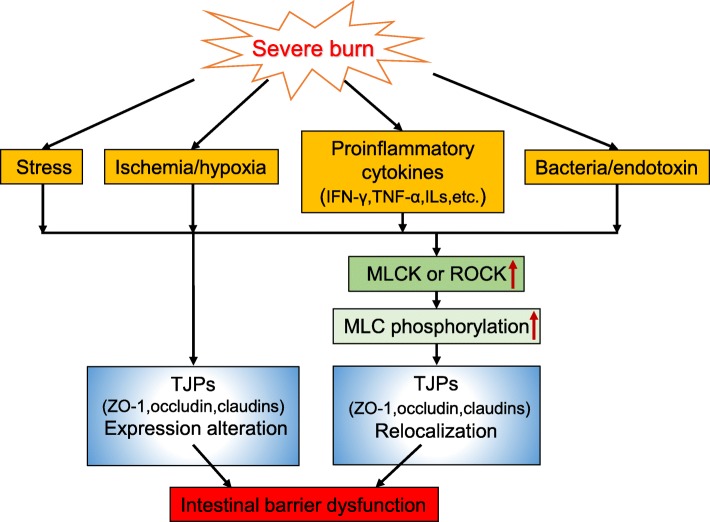
The schematic diagram illustrating the mechanisms of intestinal barrier dysfunction in severe burn injury. *IFN-γ* interferon-γ, *TNF-α* tumor necrosis factor, *ILs* interleukins, *MLCK* myosin light chain kinase, *ROCK* rho-associated protein kinase, *MLC* myosin light chain, *TJPs* tight junction proteins, *ZO-1* zonula occludens

A large number of animal and clinical studies have demonstrated that the intestinal mechanical barrier is often disrupted by severe burn injury [26–29]. The reason for intestinal mechanical barrier dysfunction induced by severe burn injury is intricate and complex. Many factors such as neuroendocrine mediators, hypoxia/ischemia, complement activation, oxygen free radicals, inflammatory mediators, proinflammatory cytokines, and other mediators released in the stress response are directly or indirectly involved in the occurrence and development of intestinal mechanical barrier dysfunction associated with severe burn injury (Fig. 1). In addition, the disruption of intestinal mechanical barrier is also closely associated with burn shock, inflammation, infection, immune disturbance, hypermetabolism, sepsis, mutiple organ dysfunction syndrome, etc. [30, 31].

It has already been reported that the intestinal permeability is increased quickly and significantly in both patients and animals suffering from severe burn injury [32, 33]. Once the intestinal permeability is increased, the luminal bacteria and/or endotoxins translocate across the disrupted intestinal barrier, and then rapidly spread to distant organs such as the liver, spleen, lungs, and even the blood via the portal vein and/or the lymphatic system. The endotoxin translocation can arise as early as 15 min post-burn and reach the peak within 6 h [34]. Our previous studies have shown that in mice subjected to a 30% total body surface area (TBSA) full-thickness burn, the ileal permeability is markedly increased at 1 h, peaks at 6 h, and still significantly higher than control level at 24 h post-burn [26, 27, 35]. Similarly, in mice suffering from 30% TBSA full-thickness scald, the intestinal permeability is significantly increased at 2 h and reaches the peak at approximately 4–6 h, whereas the histological structure of intestinal mucosa is not changed at 2 h after injury [36, 37]. Thus, it is worthy to note that after severe burn injury, the increase of intestinal permeability does not synchronize with the damage of mucosal histological structure although the increased intestinal permeability is closely associated to the changes of mucosal histological structure. The intestinal hyperpermeability induced by severe burn injury can arise earlier than the mucosal histological changes. This phenomenon may be mainly attributed to the dysfunction of intestinal epithelial TJ barrier, but the impairment of mucosal histological structure definitely aggravates the increased permeability.

### Factors contributing to the burn-induced intestinal barrier dysfunction

#### Stress

After severe burn injury, a variety of stressors such as mental stimulation, local tissue damage, ischemia/hypoxia, inflammation, and surgical operation can enhance the activities of both hypothalamic-pituitary-adrenal axis and sympathetic nerve system, leading to severe systemic stress responses. The stress may play an important role in the intestinal barrier dysfunction and hyperpermeability in some diseases including burn injury [38, 39]. An animal experiment has demonstrated the intestinal permeability, bacterial translocation, and proinflammatory cytokines such as interferon-γ (IFN-γ) are significantly increased by stress stimulation in C57BL/6 J mice, but not in the severe combined immunodeficiency (SCID) and IFN-γ-deficient mice [40], indicating that the stress-induced intestinal barrier dysfunction depends on the presence of CD4 + T lymphocytes and IFN-γ. In addition, inhibiting the phosphorylation of MLC by MLCK inhibitor ML-7 can significantly alleviate the stress-induced intestinal barrier dysfunction, suggesting that the MLCK-mediated MLC phosphorylation is involved in the stress-induced intestinal barrier dysfunction. Similarly, other investigators have also shown that acute stress can decrease the mRNA expression of TJ proteins (TJPs) ZO-2 and occludin, which in turn leads to intestinal barrier dysfunction [41].

#### Ischemia/hypoxia and ischemia-reperfusion injury

Extensive burn injuries cause extravasation of plasma, and then lead to hypovolemic shock, resulting in the ischemia and hypoxia of tissues. It is well recognized that ischemia/hypoxia is the most fundamental reason for systemic damages in the early stage of severe burn injury. Thus, ischemia/hypoxia is believed to play an important role in the pathogenesis of intestinal barrier dysfunction induced by severe burn injury. Previous studies have revealed that systemic or intestinal ischemia/reperfusion causes the intestinal barrier disruption, resulting in the increase of paracellular permeability [42–44]. It has been reported that the intestinal permeability is significantly increased 30 min after hemorrhagic shock, and the luminal bacteria translocate to the mesenteric lymph nodes, liver, and spleen [45]. The intestinal barrier disruption induced by hemorrhagic shock is accompanied by the activation of actin depolymerizing factor/cofilin, the increase of G-actin, and the decreases of F-actin, ZO-1, and claudin-3 in intestinal epithelia. Our previous study has shown that in mice subjected to 30% TBSA full-thickness flame burn, the intestinal permeability is significantly increased during the shock stage of burn and simultaneously accompanied by the decrease and redistribution of ZO-1 and the rearrangement of cytoskeleton F-actin [26]. Similarly, the intestinal permeability is significantly increased in severely scalded mice and also accompanied by the decreased expression and redistribution of both ZO-1 and occludin [35, 46]. In addition, our previous *in vitro* study has revealed that hypoxia alone can increase the permeability of cultured intestinal epithelial cell monolayers and cause the morphological changes of ZO-1 [47]. Thus, ischemia and/or hypoxia arising in the early stage of severe burn injury causes intestinal barrier dysfunction by disrupting the epithelial TJ, leading to the increase of permeability.

It has been well recognized that reperfusion after ischemia can produce large amounts of free radicals. The free radicals, on the one hand, can directly interact with the polyunsaturated fatty acid of the phospholipid membrane to form lipid peroxides, causing the impairment of intestinal epithelial cells. They, on the other hand, can also activate complements, mediate the release of inflammatory mediators, and induce cell apoptosis, leading to the disruption of the intestinal epithelial barrier. The free radicals have been demonstrated to induce the depolymerization and rearrangement of cytoskeleton F-actin and disrupt the intestinal epithelial barrier, in which PKC-λ activation is involved [48]. Moreover, reperfusion following ischemia not only damage the structure of intestinal epithelial TJ, but also alter the expression and localization of TJPs. Animal studies have shown that at 1 h post-reperfusion, the intestinal permeability is increased significantly while the histological structure of mucosa is obviously damaged as evidenced by the appearance of denuded villi, focal necrosis, ulcer, and bleeding. Meanwhile, the intestinal epithelial TJ is disrupted, and the expression and localization of ZO-1, occludin, and claudin-1, claudin-2, claudin-3, claudin-4, and claudin-5 are remarkedly changed following intestinal ischemia/reperfusion injury [49, 50].

#### Proinflammatory cytokines

After severe burn injury, large amounts of proinflammatory cytokines are released from both gastrointestinal tract and other organs [51]. Among these proinflammatory cytokines, IFN-γ, tumor necrosis factor-α (TNF-α), interleukin-1β (IL-1β), and IL-6 are believed to be the most important in inducing the intestinal epithelial barrier dysfunction and leading to increased intestinal permeability.

##### IFN-γ

Many previous *in vivo* and *in vitro* studies have confirmed that IFN-γ alone or in combination with other cytokines can induce intestinal epithelial barrier dysfunction, which is mainly associated with the altered expression and/or localization of TJPs in intestinal epithelial cells. IFN-γ has been reported to activate Rho-GTPase, upregulate the expression of Rho-associated protein kinase (ROCK), and induce the endocytosis of TJPs and the relocalization of occludin, claudin-1, and JAM-A via the RhoA/ROCK pathway, resulting in the intestinal epithelial barrier dysfunction [52]. Our *in vitro* studies have demonstrated that by inducing the expression of TNF receptor 2 (TNFR2) in the intestinal epithelial cells, IFN-γ synergizes with TNF-α to induce the expression of MLCK protein and to activate the MLCK-MLC phosphorylation pathway, which in turn results in the relocalization of TJPs ZO-1, occludin, and claudin-1, leading to the disruption of intestinal epithelial barrier function [53]. In addition, IFN-γ also downregulates the expressions of both ZO-1 and occludin by activating adenosine monophosphate-activated protein kinase, resulting in intestinal epithelial barrier dysfunction [54]. Moreover, IFN-γ induces the intestinal barrier dysfunction *in vitro* as assessed by the drop of transepithelial electrical resistance and the increases of both permeability and *Escherichia coli* transcytosis. However, inhibiting phosphatidylinositol 3′-kinase (PI-3 K) activity with LY294002 and wortmannin, the PI-3 K inhibitors, completely blocks the intestinal barrier dysfunction evoked by IFN-γ, suggesting the involvement of PI-3 K in the IFN-γ-induced intestinal barrier dysfunction [55]. Accordingly, IFN-γ disrupts intestinal epithelial barrier function via multiple signal transduction pathways.

##### TNF-α

A large number of previous studies have documented that TNF-α, alone or with other proinflammatory cytokines, can induce intestinal epithelial barrier disruption both *in vivo* and *in vitro* [56–59]. The underlying mechanisms involved in the TNF-α-induced intestinal epithelial barrier dysfunction include the apoptosis of intestinal epithelial cells, changes of the lipid composition in cell membrane, activation of MLCK by Ca^2+^-calmodulin, induction of MLC phosphorylation by upregulated MLCK protein expression, and inhibition of TJP expression, among which the MLCK-mediated MLC phosphorylation pathway is considered to play a critical role in the TNF-α-induced intestinal epithelial barrier dysfunction. It has been revealed that upon activated by TNF-α, nuclear factor of activated B cells protein kinase (NF-κB) binds to the NF-κB motif in the MLCK gene promoter, which initiates the MLCK gene transcription and then upregulates the MLCK protein expression, subsequently leading to intestinal epithelial barrier dysfunction [60]. Our previous *in vitro* studies have demonstrated that TNF-α acts synergistically with IFN-γ to induce intestinal epithelial barrier dysfunction in a dose-dependent manner, which is mediated by TNFR2 but not TNFR1. The molecular mechanism involves the activation of activator protein-1, which induces MLCK gene transcription, the upregulation of MLCK protein expression and MLC phosphorylation, and the relocalization of ZO-1, occludin, and claudin-1, resulting in the disrupted intestinal epithelial barrier [53, 61, 62]. In addition, our recent *in vitro* studies have demonstrated that hypoxia-inducible factor-1α (HIF-1α) is involved in the intestinal epithelial barrier dysfunction, the alteration of TJPs, and the increase of permeability induced by the synergistic action of TNF-α and IFN-γ, as evidenced by that the TNF-α and IFN-γ-induced intestinal epithelial barrier dysfunction is alleviated by both 3-(5'-hydroxymethyl-2'-furyl)-1-benzylind azole (YC)-1 and oligomycin, the specific chemical inhibitors of HIF-1α [63, 64]. We have also shown that lymphotoxin-like inducible protein that competes with glycoprotein D for herpes virus entry on T cells (LIGHT), a superfamily member of TNF (TNFSF14), disrupts the intestinal epithelial barrier by activating the MLCK-MLC phosphorylation pathway, which is mediated by the lymphotoxin β receptor (LTβR) belonging to the TNF receptor superfamily and the caveolin-1-dependent endocytosis of occludin [65]. The caveolin-1-dependent endocytosis of occludin is reported to be very important in the TNF-α-induced alteration of TJ [57].

##### Interleukins

Among the numerous ILs, IL-1β is the most studied in the regulation of intestinal epithelial barrier function. IL-1β has been well documented to be able to disrupt the intestinal epithelial barrier, leading to increased permeability [66–69]. The intestinal barrier disruption induced by IL-1β is most likely associated with the decreased expression and the relocalization of TJP occludin. The upregulated MLCK gene transcription triggered by NF-κB activation is also part of the mechanism by which IL-1β disrupts the intestinal epithelial barrier. It has been reported that the IL-1β-evoked increase of intestinal epithelial permeability is mediated by activation of extracellular signal-regulated kinases 1/2 (ERK1/2) signaling pathway and that inhibition of ERK1/2 activity inhibits the IL-1β-induced increase in intestinal epithelial permeability. The IL-1β-induced activation of ERK1/2 pathway leads to a downstream activation of nuclear transcription factor Elk-1. The activated Elk-1 translocates to the nucleus and binds to the *cis*-binding motif on MLCK promoter region, triggering MLCK gene activation, MLCK mRNA transcription, MLCK protein synthesis, and MLCK catalyzed opening of the intestinal epithelial TJ [70]. The p38 MAPK is also reported to be involved in the process of MLCK gene transcription induced by IL-1β. The IL-1β-induced increase of intestinal epithelial permeability is accompanied by the activation of p38 MAPK. The activated p38 MAPK then induces the phosphorylation and activation of activating transcription factor-2 (ATF-2), a substrate of p38 MAPK. The activated ATF-2 translocates to the nucleus where it attaches to its binding motif on the MLCK promoter region, leading to the activation of MLCK promoter activity and gene transcription. The IL-1β-induced activation of MLCK promoter and MLCK mRNA transcription is prevented by small interfering RNA induced silencing of ATF-2, or mutation of the ATF-2 binding motif on the MLCK promoter region [71]. Therefore, multiple signal transduction pathways are involved in the intestinal epithelial barrier dysfunction evoked by IL-1β, but the upregulation of MLCK gene transcription is the most critical.

Among the other ILs, IL-4 has been reported to disrupt intestinal barrier function via the PI-3 K signaling pathway [72], whereas the effect of IL-6 on intestinal barrier function is still controversial. On the one hand, it has been shown that IL-6 can induce intestinal barrier dysfunction and increase the permeability and that inhibition of IL-6 or knockout of the IL-6 gene can prevent the damage of intestinal mucosa and the increase of permeability in intestinal ischemia/reperfusion injury, sepsis, and hemorrhagic shock [73, 74]. An *in vitro* study has shown that IL-6 can only cause the increase of intestinal permeability to ions but not to large-molecule materials. This characteristic of IL-6 on the intestinal barrier is associated with the induced expression of claudin-2, but not ZO-1, ZO-2, occludin, JAM-1, or claudin-1, claudin-3, and claudin-4, in which the MEK/ERK and PI-3 K/Akt signal pathways are involved [75]. On the other hand, however, IL-6 has been reported to protect intestinal epithelial barrier function, and the potential mechanism may be related to the IL-6-induced upregulation of keratin-8 and 18 in intestinal epithelial cells [76]. Similarly, an *in vivo* study has demonstrated that IL-6 neutralizing antibody treatment can significantly reduce the ileal mucosa damage, bacterial translocation to the mesenteric lymph node, and relocalization of ZO-1 and occludin following the combined insult of ethanol exposure and burn injury; however, IL-6 gene knockout has no similar effects [77]. These findings suggest that maintaining the appropriate amount of IL-6 may be helpful to restore intestinal barrier function in inflammation, whereas complete loss of IL-6 seems not to be beneficial to maintain the intestinal barrier. In addition, IL-13 has also been reported to play an important role in the pathogenesis of intestinal barrier dysfunction. A recent study has shown that IL-13 induces the increased permeability of intestinal epithelium to ions in a dose-dependent manner, in which the IL-13-induced increase and redistribution of claudin-2 via signal transducers and activators of transcription 6 (STAT6) signaling pathway is involved [78].

#### Bacteria and endotoxins

Severe burn injury has been reported to be able to alter intestinal microbiota composition in both animals and patients, resulting in the intestinal microbiota dysbiosis [79–81]. The intestinal flora disturbance may increase the conditional pathogenic bacteria. Among the bacteria, enteropathogenic *Escherichia coli* (EPEC) is the most studied in the regulation of intestinal epithelial barrier function. Previous studies have determined that EPEC is capable of inducing the intestinal epithelial barrier dysfunction [82–84]. Utilizing its type III secretion system (T3SS), EPEC injects pathogenic effector proteins such as EspF, EspG, EspH, and Map into the intestinal epithelial cells, causing the cytoskeleton collapse and relocalization of TJPs. The resulting compromised barrier and increased intestinal permeability may be responsible for the clinical symptoms of EPEC infection. Some *in vivo* studies have revealed that EPEC infection causes the disruption of the intestinal barrier and the relocalization of TJPs. The disruptive effect of EPEC on the intestinal epithelial barrier is dependent on the pathogenic effector protein EspF [85–87]. Notably, the EPEC-induced intestinal barrier dysfunction is also related to the activation of the MLCK-MLC phosphorylation pathway, because that inhibition of MLCK with ML-9, a specific chemical inhibitor of MLCK, can prevent the intestinal barrier defects induced by EPEC infection [88]. The PKCε signaling pathway is also reported to be involved in the intestinal epithelial barrier disruption induced by EPEC infection [89]. In addition, enteroinvasive *Escherichia coli* (EIEC) has also been demonstrated to evoke the relocalization and reduced expression of ZO-1, occludin, claudin-1, and JAM-1 in the intestinal epithelial cells, resulting in the intestinal epithelial barrier dysfunction [90].

A huge number of studies have determined the disruptive effect of endotoxin on intestinal epithelial barrier both *in vivo* and *in vitro*. On the one hand, endotoxin disrupts the intestinal barrier by inducing a variety of cells to produce proinflammatory mediators such as TNF-α and IL-1β. On the other hand, endotoxin also directly impairs intestinal epithelial cells to cause the barrier dysfunction. An *in vivo* study has shown that intestinal permeability and bacterial translocation is significantly increased and the intestinal epithelial TJs are obviously opened in lipopolysaccharide (LPS)-challenged rats. This disruptive effect of LPS on intestinal barrier depends on MLCK, because that inhibiting MLCK activity with ML-7, a specific inhibitor of MLCK, can alleviate the intestinal barrier dysfunction and bacterial translocation in rats challenged with LPS [91]. Moreover, LPS has been reported to cause the relocalization of ZO-1, occludin, and claudin-1 and claudin-4 and reduce the expression of ZO-1, in which c-Src, toll-like receptor 4 (TLR4), and LPS binding protein are involved [92]. Our latest in vitro study has revealed that LPS also induces epithelial barrier disruption by activating Nod-like receptor protein 3 (NLRP3) inflammasome and autophagy in Caco-2 intestinal epithelial monolayers [93].

### Mechanisms involved in the burn-induced intestinal barrier dysfunction

#### Alteration of TJPs

It is well known that the structural basis of intestinal epithelial barrier integrity is TJ and its associated proteins such as ZOs, occludin, and claudins. However, the changes in intestinal epithelial TJPs after severe burn injury are not well defined so far. In general, there are mainly two aspects about the changes in intestinal epithelial TJPs after severe burns: one is the relocalization or redistribution of the TJPs in intestinal epithelial cells, and the other is the altered expression of TJPs. Our previous study has demonstrated that there is an obvious relocalization of ZO-1, occludin, and claudin-1 in the ileal mucosa of mice inflicted with 30% TBSA full-thickness flame burns [94]. Other investigators have also shown that the protein expression of occludin in intestinal epithelial cells is not significantly changed in rats subjected to a 30% TBSA burn, but the relocalization of occludin is evident after burn injury. There is a clearly decreased expression and relocalization of occludin in the ileum of burned rats combined with *Enterococcus faecalis* infection [95]. It has been reported that the expressions of ZO-1 and occludin in intestinal epithelia are decreased to the lowest at 6 h post-burn, with reductions of 68% and 43% respectively, and still significantly lower than control level at 24 h post-burn [46]. Furthermore, simple hemorrhagic shock can also induce the decreased expression and relocalization of ZO-1 and claudin-3, leading to intestinal TJ barrier dysfunction and bacterial translocation [45]. Therefore, both the decrease and relocalization of TJPs in intestinal epithelial cells after severe burn injury are bound to impair the structure and function of intestinal epithelial TJ, resulting in barrier disruption and hyperpermeability.

#### Role of MLC phosphorylation

It is well established that the TJs are connected to cytoskeleton actin through ZOs [22, 96–98]. Thus, the integrity of the intestinal TJ barrier also depends on the structural assembly and functional status of the intestinal epithelial cytoskeleton actin as well as the interaction between myosin and actin. A critical step in the regulation of epithelial TJ permeability may be myosin ATPase-mediated contraction of the perijunctional actomyosin ring and subsequent physical tension on the TJ [99], to which MLC phosphorylation is critical. It is well known that MLC phosphorylation is regulated by both MLCK and MLC phosphatase (MLCP) [100], which is more commonly called myosin phosphatase. On the one hand, MLCK phosphorylates MLC, resulting in an increase of MLC phosphorylation. On the other hand, MLCP dephosphorylates MLC, leading to a reduction of MLC phosphorylation. It has been reported that the induction of MLC phosphorylation in intestinal epithelial cells alone is sufficient to change the TJPs ZO-1 and occludin, leading to the increased permeability of the intestinal epithelial TJ [101]. Our previous animal studies have shown that the remarkably increased intestinal permeability and the redistribution of TJPs following severe burn injury are accompanied by the significantly increased MLC phosphorylation [26, 27, 94]. Similarly, our *in vitro* studies have also revealed that the barrier disruption caused by burn sera or hypoxia is accompanied by a significant increase of MLC phosphorylation in cultured intestinal epithelial cell monolayers [47, 94]. Thus, the increased MLC phosphorylation may be a central molecular mechanism involved in the severe burn-induced intestinal barrier disruption and hyperpermeability.

#### Role of MLCK

It is well known that MLCK, a member of the serine/threonine protein kinase family, is a Ca^2+^/calmodulin-dependent kinase responsible for the phosphorylation of a specific serine in the N-terminus of MLC. Upon activated by upstream signals such as Ca^2+^, histamine, bradykinin, free radicals, and proinflammatory cytokines, MLCK can induce the phosphorylation of serine 18/threonine 19 of MLC in nonmuscle cells. It has been well documented that MLCK is a central determinant in the intestinal epithelial barrier dysfunction under various pathological conditions, and the underlying mechanism involves the induction of MLC phosphorylation. Based on the reported studies, it is believed that MLCK also plays a critical role in intestinal barrier dysfunction after severe burn injury. Some previous studies have shown that MLCK is critical to intestinal epithelial barrier dysfunction, increased permeability, and the relocalization of TJPs ZO-1, occludin, and claudins induced by a number of pathophysiological conditions associated with severe burns such as stress, shock, ischemia/hypoxia, inflammation, and infection [5, 39, 47, 97, 101–104]. The previous animal studies have revealed that MLCK chemical inhibitor ML-9 or peptide inhibitor PIK could significantly alleviate the barrier dysfunction, the changes of TJPs, the increase of MLC phosphorylation, and the hyperpermeability of intestinal epithelia in mice subjected to severe burns [94, 105]. Similarly, in MLCK knockout mice inflicted with 40% TBSA full-thickness burn, there was only slight elevation of intestinal permeability at 4 h after the injury, without obvious damage to the intestinal mucosa [106]. Therefore, after severe burn injury, MLCK upregulates the phosphorylation of MLC in intestinal epithelial cells, thereby leading to the barrier dysfunction and hyperpermeability.

#### Role of ROCK

Rho, which belongs to the Ras superfamily of low molecular weight GTPases, is also an important regulator of the intestinal epithelial barrier. It regulates the permeability of the intestinal barrier by mainly affecting the interaction between actin and myosin in epithelial cells. The downstream effector of RhoA is ROCK, a serine/threonine protein kinase. The activated ROCK phosphorylates the myosin phosphatase target subunit (MYPT), which is also called the myosin-binding subunit (MBS) of myosin phosphatase, and consequently inactivates myosin phosphatase, thereby increasing the phosphorylation of MLC. In other words, the activation of ROCK results in an increase of MLC phosphorylation. At present, there are few data about the involvement of ROCK in post-burn intestinal barrier dysfunction. However, some studies have determined that ROCK is involved in the intestinal epithelial barrier disruption caused by proinflammatory cytokines, inflammation, and bacteria [52, 107, 108]. For example, ROCK has been reported to mediate the bacteria-induced increase of intestinal epithelial permeability and the decreased expression and relocalization of claudin-4 and claudin-5 [108]. Our previous study has demonstrated that intestinal permeability, ROCK protein expression, and MLC phosphorylation are elevated in mice subjected to 30% TBSA full-thickness burns. However, intraperitoneal injection of ROCK-specific inhibitor Y-27632 immediately after the injury can significantly alleviate the severe burn-induced increases of intestinal permeability and MLC phosphorylation [27]. Similarly, burn sera can induce barrier dysfunction and upregulate ROCK protein expression and MLC phosphorylation in cultured intestinal epithelial cell monolayers, whereas inhibiting ROCK activity with Y-27632 can also alleviate the burn sera-induced barrier dysfunction and the increase of MLC phosphorylation in cultured intestinal epithelial cell monolayers [109]. Therefore, ROCK is believed to be involved in the severe burn-induced intestinal barrier dysfunction and hyperpermeability by inducing MLC phosphorylation.

### Therapeutic strategies for the burn-induced intestinal barrier dysfunction

It is well recognized that the treatment of post-burn intestinal barrier dysfunction is an important part of the burn treatment and directly related to the level of comprehensive treatment of severe burns. The factors or pathophysiological processes affecting intestinal barrier function after severe burns are multiple and complex. Thus, the appropriate therapeutic measures should correspondingly be taken to every critical pathophysiological process, such as stress, shock, ischemia/hypoxia, inflammation, infection, and surgical operation. Many clinical and experimental studies in the past have suggested that taking some positive and reasonable measures is beneficial to the intestinal barrier in the early stage of severe burns. These measures include positive anti-shock to improve the oxygen supply to organs including intestine, control of inflammation and infection, wound management, early enteral nutrition, immunonutrition, ecoimmunonutrition, and supplementation of some special nutrients such as glutamine and arginine [2, 30, 31].

In addition to the aforementioned therapeutic measures, an animal study has recently demonstrated that zinc finger aspartate-histidine-histidine-cysteine (DHHC) domain-containing protein-21 (ZDHHC21), a particular palmitoyl acyltransferase, mediates the intestinal epithelial hyperpermeability in mice subjected to a 40% TBSA full-thickness scald injury [110]. The thermal injury-induced intestinal barrier dysfunction is significantly attenuated in mice with genetic ablation of ZDHHC21 or by intraperitoneal injection of 2-bromopalmitate, a pharmacological inhibitor of palmitoyl acyltransferases, suggesting that targeting palmitoyl acyltransferase ZDHHC21 can effectively attenuate the intestinal epithelial barrier disruption caused by severe burn injury. Moreover, endoplasmic reticulum stress (ERS)-autophagy pathway has recently been demonstrated to be associated with intestinal epithelial TJ barrier dysfunction induced by severe burn injury [35]. Inhibiting ERS or autophagy with specific inhibitor can significantly ameliorate the severe burn-induced intestinal TJ barrier dysfunction [111]. It is implied that blocking ERS-autophagy pathway may be beneficial to restoring intestinal epithelial TJ barrier dysfunction induced by severe burn injury. Furthermore, it is very interesting that mesalazine, which is also known as 5-aminosalicylic acid and a common clinical anti-inflammatory drug used to treat inflammatory bowel disease, has recently been reported to be able to treat the intestinal barrier disruption resulting from burn injury [112]. Treatment with mesalazine after burn injury prevents the burn-induced increase of intestinal permeability, normalizes the levels of pro-inflammatory cytokines, and restores the expression of TJPs claudin-4 and occludin, which indicates that mesalazine can potentially be used as the therapeutic drug for intestinal barrier disruption induced by severe burn injury.

With the deepening understanding of the regulatory mechanisms of intestinal epithelial TJ, new therapeutic strategies aiming at the regulation of intestinal epithelial TJ may be the direction for the prevention and treatment of post-burn intestinal barrier dysfunction, especially the measures targeting at MLC phosphorylation. This may be more important for controlling increased permeability when the histological structure of intestinal mucosa has not yet been damaged obviously. The previous animal studies have already demonstrated that inhibiting MLCK and ROCK activity by using specific inhibitors to reduce MLC phosphorylation in intestinal epithelial cells can significantly alleviate the severe burn-induced intestinal barrier dysfunction and hyperpermeability [27, 94, 105]. Moreover, it has been reported that both phosphodiesterase inhibitor pentoxifylline and p38 MAPK inhibitor SB203580 can reduce intestinal permeability and protect intestinal barrier function in severe burn injury, the underlying mechanism of which is proved to be mediated by inhibiting the MLCK-mediated upregulation of MLC phosphorylation [36, 113]. Another *in vivo* study has also revealed that inhibiting MLCK-mediated MLC phosphorylation by MLCK inhibitor ML-7 can prevent the increase of intestinal permeability, opening of TJ, bacterial translocation, and intestinal inflammatory response in rats challenged with endotoxin [91]. Most notably, the latest study has revealed that divertin, a domain-binding small molecule that blocks MLCK1 recruitment without inhibiting enzymatic function, is capable of blocking acute, TNF-induced MLCK1 recruitment as well as downstream MLC phosphorylation, barrier loss, and diarrhea both in vitro and in vivo, suggesting that MLCK1 diversion can reverse intestinal epithelial barrier loss [114]. Thus, although there are still many issues that require to be further clarified, such as the effectiveness, safety, and feasibility on clinical applications, the therapeutic strategies targeting at MLC phosphorylation pathway may be the direction of future efforts in the prevention and treatment of intestinal barrier dysfunction after severe burn injury.

## Conclusions

The integrity of intestinal barrier function is essential to maintain the homeostasis of the intestinal mucosa. However, the intestinal barrier is disrupted after severe burn injury. A broad range of pathogenic factors is known to induce the disruption of epithelial TJ barrier, thereby leading to the intestinal hyperpermeability. The mechanism of intestinal barrier disruption induced by severe burn injury is extremely complex, involving numerous signaling molecules and related pathways. A better basic understanding of the mechanism might be helpful for the prevention or treatment of intestinal barrier disruption following severe burn injury.

## Data Availability

Not applicable

## Abbreviations

Elk-1: Ets-like gene 1
EPEC: Enteropathogenic *Escherichia coli*
ERK: Extracellular signal-regulated kinase
ERS: Endoplasmic reticulum stress
IFN-γ: Interferon-γ
IL: Interleukin
JAM: Junctional adhesion molecule
LIGHT: Lymphotoxin-like inducible protein that competes with glycoprotein D for herpes virus entry on T cells
LPS: Lipopolysaccharide
MAPK: Mitogen-activated protein kinases
MLC: Myosin light chain
MLCK: Myosin light chain kinase
NLRP3: Nod-like receptor protein 3
PDZ: Postsynaptic density protein-95/Discs-Large/zonula occludens-1
PI-3 K: Phosphatidylinositol 3′-kinase
PKC: Protein kinase C
ROCK: Rho-associated protein kinase
SCID: Severe combined immunodeficiency
STAT6: Signal transducers and activators of transcription 6
TBSA: Total body surface area
TJ: Tight junction
TJP: Tight junction protein
TLR4: Toll-like receptor 4
TNFR: Tumor necrosis factor receptor
TNF-α: Tumor necrosis factor-α
ZDHHC21: Zinc finger aspartate-histidine-histidine-cysteine domain-containing protein-21
ZO: Zonula occludens
